# Identification and Characterization of Resistance to Rust in Lentil and Its Wild Relatives

**DOI:** 10.3390/plants12030626

**Published:** 2023-01-31

**Authors:** Eleonora Barilli, Diego Rubiales

**Affiliations:** Institute for Sustainable Agriculture, CSIC, Avda. Menéndez Pidal s/n, 14004 Córdoba, Spain

**Keywords:** crop wild, plant breeding, *Lens*, quantitative resistance, hypersensitive response, *Uromyces viciae-fabae*, screening, differentials set

## Abstract

Lentil rust is a major disease worldwide caused by *Uromyces viciae-fabae*. In this study, we screened a large germplasm collection of cultivated lentils (*Lens culinaris* ssp. *culinaris*) and its wild relatives, both in adult plants in the field with a local rust isolate during 2 seasons and in seedlings under controlled conditions with four fungal isolates of worldwide origin. The main results from our study were the following: (1) a significant number of accessions with resistance based on hypersensitive reaction (reduced Infection Type (IT)) were identified in cultivated lentil and in *L. ervoides*, *L. nigricans* and *L.c. orientalis.* The IT scores showed a clear isolate-specific response suggesting race-specificity, so each fungal isolate might be considered a different race. Resistance was identified against all isolates what might be the basis to develop a standard differential set that should be a priority for rust definition and monitoring. (2) Interestingly, although at lower frequency than in *L. ervoides* and *L. nigricans*, the hypersensitive response was also observed within cultivated lentil, with accession 1561 (*L.c. culinaris*) displaying resistance to the four isolates making this accession a valuable ready-to-use resource for lentil resistance breeding. Resistance to all other rust isolates was also available within *L.c. culinaris* in an isolate-specific manner. Accession 1308 (*L. ervoides*) showed resistance against all isolates tested, as well as a reduced number of accessions belonging to other wild *Lens* species. (3) In addition, our screenings allowed the identification of several accessions with partial resistance (reduced Disease Severity (DS) despite high IT). Adult Plant Resistance resulting in reduced severity in adult plants in the field, despite high susceptibility in seedlings, was more frequently identified in *L.c. culinaris*, but also in *L. nigricans* and *L.c. orientalis*.

## Highlights

► Hypersensitive, partial and adult plant resistances have been identified within *L.c. culinaris* accessions, enabling their immediate use in lentil resistance breeding
► Additional valuable sources of resistance have been identified in related species of the primary and secondary gene pools, crossable with cultivated lentils making feasible the transfer of rust resistance genes.

## 1. Introduction

Lentil (*Lens culinaris* Medik.) is an annual food legume cultivated over 5.5 million hectares [1]. Average yields are small (word average 1000 kg/ha) which might be ascribed to biotic and abiotic constraints and to the fact that lentil is generally produced under low input conditions [2,3]. Rust, caused by the fungus *Uromyces viciae-fabae* (Pers.) Schröt (syn. *U. fabae* (Pers.) de Bary) is regarded as one of the most important foliar diseases of lentil, widespread globally, with reported yield losses ranging from 25 to 100% [2,4,5]. Rust can be controlled by a number of fungicides but economic factors must be taken into consideration [6,7].

Breeding for rust resistance is regarded as the most cost-efficient method for rust control in legume crops [5,8]. Resistance to rust has been reported in lentil [9,10,11], even when the variable levels of detail hinder the comparison of results due to a lack of information details regarding the inoculation conditions, inoculum identity or the resistance components assessed. The few genetic studies available suggest monogenic control [12,13,14,15] that does not preclude the existence of polygenic resistance; variable levels of incomplete resistance have also been reported [9,11] although its inheritance is not yet studied. Stability and durability of resistance is one of the most important concerns for breeders, which reinforces the need to search and characterize additional sources of resistance.

Rust resistance breeding is hampered by insufficient knowledge of physiological specialization in the pathogen [16], which deserves urgent monitoring. In fact, even information on the causal agent is often misleading as *Uromyces viciae-fabae* is today acknowledged to be a complex species in which crop specialization is occurring [17,18,19]. The clear-cut crop specialization of isolates from faba bean (*Vicia faba*), common vetch (*Vicia sativa*) and lentil allowed subdivision of *U. viciae-fabae* into at least *U.v-f* ex *Vicia faba*, *U.v-f* ex *V. sativa* and *U.v-f* ex *L. culinaris*. Additionally, under favorable weather conditions, crop failure may also occur caused by pea rust incited by the fungus *U. pisi* [20,21].

Lentil suffers from relatively low genetic diversity due to a genetic bottleneck created during domestication with selection for a small number of traits [22]. This reinforces the interest in the incorporation of genetic diversity available in wild relatives where resistance to rust has been reported [23,24] such as *L. culinaris* ssp. *orientalis*, *L. culinaris* ssp. *odemensis* or *L. ervoides* which can be hybridized with cultivated lentil [25,26], making feasible the transfer of rust resistance genes.

The objectives of the present work were to identify and characterize additional sources of resistance to rust in lentil germplasm in its wild relatives and to test their stability in the field in different seasons and in seedlings under controlled conditions against contrasting isolates of the pathogen.

## 2. Materials and Methods

### 2.1. Lens sp. Germplasm Origin

This study used a worldwide germplasm collection containing 523 accessions kindly provided by CRF (Centro Nacional de Recursos Fitogenéticos, Spain), USDA-ARS (Department of Agriculture, USA) and ICARDA (International Centre for Agricultural Research in the Dry Areas, Syria). The collection represents the *Lens* genus in taxonomy, including 429 accessions of *L. culinaris* ssp. *culinaris*; 31 of *L. culinaris* ssp. *orientalis*; 5 of *L. culinaris* ssp. *odomensis*; 21 of *L. ervoides*; 2 of *L. lamottei*; and 34 of *L. nigricans* (Supplementary Table S1). All the accessions were multiplied at the Institute for Sustainable Agriculture—CSIC at Cordoba, Spain under field condition before the experiments.

### 2.2. Pathogen Isolate and Multiplication

Seedling experiments under controlled conditions were performed using isolates SPA, MOR, FRA and ALG of *U. viciae-fabae* ex *L. culinaris* which were previously collected from naturally infected lentil crops in Spain, Morocco, France and Algeria, respectively. The different fungal isolates were multiplied in susceptible lentils cv. Pardina in separate growth chambers (one different chamber per isolate) with filtered ventilation and conserved at −80 °C. Field experiments were only inoculated with the SPA isolate.

### 2.3. Field Experiments and Data Assessments

The *Lens* sp. collection was phenotyped over two crop seasons (2014 and 2015) at Córdoba, Spain (Table 1) using the rust susceptible lentil cv. Pardina as control check, following an alpha lattice design with three replicates. The experimental unit consisted of a single 1 m-long row per accession with 15 plants per row, separated from the adjacent row by 0.7 m, with three replications. Accessions were sown in the field by late December each year and harvested by early June, according to local practices.

Plants were artificially inoculated by mid-March, at flowering stage, by spraying with an aqueous suspension of urediospores from isolate SPA to ensure high and uniform levels of rust infection. The urediospores were suspended in tap water (6 × 10^4^ urediospores mL^−1^), to which Tween-20 (0.03%, *v*:*v*) was added as a wetting agent to reduce the surface tension of the urediospores and to obtain a homogeneous suspension. Plants were inoculated after sunset to benefit from the darkness and high relative humidity of the night. When rust development started, disease severity (DS) was assessed by a visual estimation of the percentage of plant canopy covered by rust pustules.

### 2.4. Controlled Condition Experiment and Assessments

The collection was inoculated with each of the four rust isolates separately. For this, each accession was represented by nine seedlings per round, planted 3 by 3 in 1 L pots, this repeated in four consecutive replications per isolate. Pots were placed in a randomized complete block design and seedlings were inoculated when the third leaf was completely expanded (±12 days after sowing). Two-week-old plants were inoculated by dusting with 1 mg urediospores per pot, mixed in pure talc (1:10, *v*:*v*) and incubated for 24 h at 20 °C in complete darkness and 100% relative humidity. Plants were then transferred to a growth chamber at 20 °C with a photoperiod of 14 h of light and 10 h of darkness and a light intensity of 148 µmol m^−2^ s^−1^. By 14 dpi, disease severity (DS) was visually estimated as the percentage of canopy covered by rust. In order to compare DS from different seasons and conditions, DS values from each trial were standardized by expressing each DS value as a percentage of the highest one in each location/experiment that is set at 100% (DSr) [27,28]. Infection Type (IT) was also assessed using the scale of Stakman et al. (1962) [29], where IT 0 = no symptoms, IT ; = necrotic flecks, IT 1 = minute pustules barely sporulating, IT 2 = necrotic halo surrounding small pustules, IT 3 = chlorotic halo and IT 4 = well-formed pustules with no associated chlorosis or necrosis.

All components of resistance among lentil accessions and fungal isolates were subjected to an ANOVA and mean values were separated by LSD test at *p* = 0.01. Pearson’s linear correlations between field and controlled conditions parameters were calculated.

## 3. Results

### Phenotypic Response

**Figure 1:**
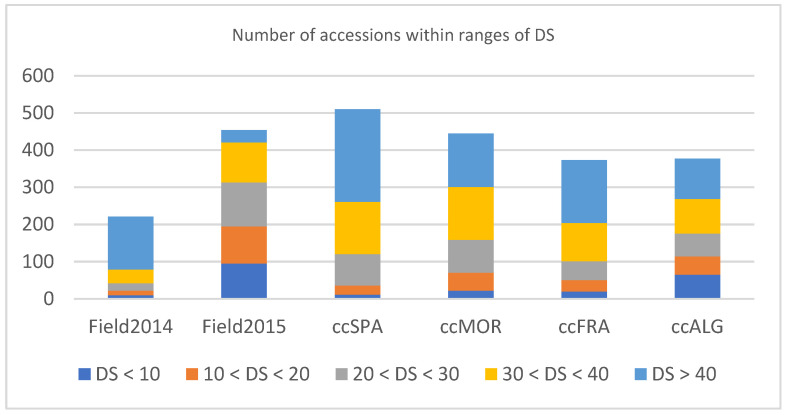
Phenotypic variation in rust response (DS) among *Lens* collection (525 accessions, different numbers studied on the different trials) after inoculation with *U. viciae-fabae* ex *L. culinaris*. Field2014 = adult plants in the field 2014; Field2015 = adult plants in the field 2015; ccSPA = seedlings under controlled conditions inoculated with isolate SPA; ccMOR = seedlings under controlled conditions inoculated with isolate MOR; ccFRA = seedlings under controlled conditions inoculated with isolate FRA; ccALG = seedlings under controlled conditions inoculated with isolate ALG.

Due to seed availability and differential germination capacity, not all accessions could be studied at all conditions, but 221 accessions were evaluated in the field in 2014, 454 in 2015, 510 in seedlings under controlled conditions against SPA isolate, 445 against MOR isolate, 373 against FRA isolate and 377 against ALG isolate, respectively (Figure 1). Large variation was identified for DS in the collections in all trials (Figure 1). Higher rust pressure was achieved in the field in 2014 than in 2015 (average DS 48% vs. 25%, respectively) which might have hidden quantitatively expressed slow rusting response that is typically better detected at moderate–low disease pressure [30]. The lower disease pressure achieved during the second season might be ascribed to the dryer conditions (342 mm of rain during first crop season compared to only 148 mm in the second season) (Table 1).

There was high variation for DS within each species, including cultivated lentil types (Table 2). However, both lower DS values (both average and range) were recorded in wild relatives although highly susceptible accessions were observed in all species. In any case, even when higher average DS values were observed in the field and under controlled conditions for cultivated lentil and the closer relative *L.c. orientalis*, accessions with high resistance were identified within both species as shown by the ranges of DS displayed in Table 2. The other way around, although average DS was low in the more distant relatives *L. ervoides*, *L. nigricans* and *L. lamottei*, susceptible accession were identified in all of them.

Adult plant responses in the field (DS%) in the two field seasons were significantly correlated (0.46, *p* < 0.001) (Table 3). DS in the field in 2015 were significantly correlated with seedling responses under controlled conditions with all isolates. However, DSfield2014 was not significantly correlated with seedling responses against isolates SPA and ALG. Seedling responses against all isolates were significantly correlated.

Hypersensitive response (IT < 3) was observed in accessions of *L.c. culinaris* in a frequency ranging from 1.4 to 3.4% depending on the isolate (Table 4). Frequency of occurrence in other species might be handled with care as lower numbers of accessions were studied, but it was more frequently identified in *L. ervoides* (9 to 21.4% of the accessions, depending on the isolate), followed by *L. nigricans* (0 to 8.3%). It was observed only against two isolates in *L.c. orientalis*, and not observed in *L.c. odemensis* or *L. lamottei*. However, this might be due to the lower number of accessions studied of these two species.

IT scores showed a clear isolate-specific response suggesting race specificity, so each isolate might be considered a different race, SPA being the most virulent one, followed by MOR and FRA, ALG being the less virulent. Hypersensitive resistance (IT < 3) was identified against all of them (Table 5 and Table 6 and Supplementary Table S1). Accessions 1308 (*L. ervoides*) and 1561 (*L.c. culinaris*) were resistant to the 4 isolates. Accession 1168 (*L.c. culinaris*) was resistant to isolates SPA, FRA and ALG, but susceptible to isolate MOR. Accessions 1515, 1559 (*L.c. culinaris*) were resistant to isolates SPA, MOR and ALG, but susceptible to isolate FRA. Accession 1599 (*L. nigricans*) was also resistant to isolates SPA, MOR and ALG, but could not be studied against isolate FRA. In addition, accession 1571 (*L. ervoides*) was resistant to isolates SPA and MOR, although it could not be studied against FRA and ALG. Accession 1145 (*L.c. culinaris*) was resistant to isolates FRA, ALG and MOR, but susceptible to isolate SPA. Accession 1413 (*L.c. culinaris*) was resistant to isolates SPA and MOR, but susceptible to FRA and ALG. Accession 1632 (*L.c. orientalis*) was resistant to isolates SPA and FRA, but susceptible to MOR and ALG. Accession 1656 (*L. nigricans*) was resistant to isolates MOR and ALG, but susceptible to SPA and FRA. Accessions 1165, 1324, 1331, 1351, 1361, 1430, 1552, 1553 (*L.c. culinaris*) were resistant to isolate ALG only, susceptible to SPA, MOR and FRA. Accessions 1288, 1470, 1471 (*L.c. culinaris*) were resistant to isolate FRA only, susceptible to SPA, MOR and ALG.

In addition to the hypersensitive response mentioned above, the screenings allowed identification of accessions with reduced rust severity in spite of a compatible interaction (high IT), fitting the definition of Partial Resistance [30,31] (Table 7 and Supplementary Table S1). There was a high variation for DSr values across accessions with high IT, but low levels of DSr at all environments (seasons and isolates) were not very frequent, suggesting isolate specificity also for DSr.

Screenings allowed identification of Adult Plant Resistance (APR) not based on hypersensitivity, with accessions 1660 (*L. nigricans*), 1613 (*L.c. orientalis*) and 1387, 1392, 1403, 1417, 1452, 1449, 1455, 1473, 1479, 1501, 1511, 1516, 1517, 1518, 1519, 1564, 1565 (*L.c. culinaris*) (Table 8) showing reduced severity in adult plants in the field (DSr < 20%), whereas they were highly susceptible in seedlings against all isolates (IT > 3, DSr > 50%).

## 4. Discussion

Lentil is an important pulse crop worldwide. However, the species suffers from relatively low genetic diversity due to a genetic bottleneck created during domestication when it underwent selection for a small number of traits [22]. This has limited the genetic variation available in the cultivated gene pool for improving important agronomic traits. This reinforces the value of exploring wild relatives as potential source of genes that might have been lost during the domestication process [32].

Resistance to rust in lentil has been identified both in cultivated lentils and its wild relatives and frequently reported to be monogenic [9,10,11,12,13,14,15,33] which does not exclude the existence of polygenic resistance. As for most cool season grain legumes [8,27], the phenotypic expression of the rust resistance reactions reported so far in lentils is poorly described. Rust resistance breeding in lentil, as in most cool season legumes, has been hampered by the relatively low investment in genetics, genomics and biotechnology of the legume crops which is impressively improving recently [34]. However, less attention has been paid to the understanding of the rust pathogen, with still little agreement on its host specialization and the existence of races [5,8], contrasting with the situation of rusts of other legumes such as common bean (*Phaseolus vulgaris*) and soybean (*Glycine max*) which have been largely studied leading to the identification of races and of resistance genes [35,36]. Surprisingly, knowledge on the existence of races or even host specialization in *U. viciae-fabae* is still very limited. Insights into the *U. viciae-fabae* genome have been initiated [37], which can help in the search for secreted proteins and effectors. However, basic knowledge of pathogenic variation is still insufficient. Race existence has been suggested within the faba bean infecting isolates [38,39] and in lentil isolates [40,41], indicating that pathogenic variation indeed might exist within the various *U. viciae-fabae* populations, but a standard differential set for race identification has not been agreed upon and currently races are not named, and their distributions are not monitored anywhere. Our IT scores showed a clear isolate-specific response suggesting race specificity, so each isolate might be considered a different race, SPA being the most virulent one, followed by MOR and FRA, ALG being the less virulent. Resistance was identified against all isolates, which might be the basis to develop a standard differential set what should be a priority for rust definition and monitoring [5]. 

In this study, we determined a significant number of accessions with resistance based on hypersensitive reaction (HR, low IT) in all *Lens* species studied. Hypersensitive response was more frequently identified in *L. ervoides* followed by *L. nigricans*. It was observed only against two isolates in *L.c. orientalis*, and not observed in *L.c. odemensis* or *L. lamottei*, but this might be due to the lower number of accessions studied of these two species. Interestingly, although at lower frequency than in *L. ervoides* and *L. nigricans*, hypersensitive response was also observed within cultivated lentil, with accession 1561 (*L.c. culinaris*) displaying resistance to the four isolates making this accession a valuable ready-to-use resource for lentil resistance breeding. Resistance to all other rust isolates was also available within *L.c. culinaris* in an isolate-specific manner. Accession 1308 (*L. ervoides*) was also resistant (low IT) to all isolates, and a number of accessions of other wild *Lens* species also displayed resistance against some of the isolates in an isolate-specific manner, calling the attention to the need to study their inheritance to discern whether novel resistance gene(s) are different from those in *L.c. culinaris*. Although we did not study the inheritance, a feasible starting hypothesis might be that they might be monogenic as is typically the case for HR. It is important to clarify that HR can be complete (IT 0) but also incomplete, allowing some sporulation and rust development (IT 1–2).

Both pre-haustorial- and post-haustorial-based types of resistance were earlier reported in lentil germplasm [10,11]. Post-haustorial resistance is typically based on hypersensitivity, whereas pre-haustorial resistance is not, and is typical in partial resistance causing a reduced DS with no host cell necrosis [42,43,44,45,46,47]. However, the use of “partial resistance” concept might be misleading as incomplete HR can often be confounded with partial resistance if not enough attention is paid to the presence/absence of macroscopically visible necrosis associated with developing rust pustules. This might be the case of reported single inheritance to rust in lentil, but we cannot draw a conclusion as these reports are often based on field screenings without detailed description of types of resistance responses but just on scales based on amount of pustules and plant damage, such as the 1–9 scale [48] without clear indications on actual presence or absence of necrosis indicative of HR. Therefore, care should be taken when interpreting published results based on different scales. A combination of a qualitative (such as IT based on presence/absence of necrosis) and a quantitative assessment (DS) should therefore be preferred for any rust screening to identify both partial and hypersensitive resistances to rust, as is nowadays commonly practiced [27,46,49]. Our screenings allowed identification of accessions with partial resistance (reduced DS in spite of high IT) [30,31], but this was not very frequent.

Race non-specific adult plant resistance (APR) associated with slow rusting has frequently been exploited in wheat [50,51,52]. APR is believed to be more durable for successful long-term rust control [53,54] as it is generally not affected by race, and keeps the disease below the threshold level and decreases the chances of selection of new pathotypes. APR has also been identified in a range on legume crops against their rust [55,56] plant resistance. We identified accessions with such adult plant resistance, showing reduced severity in adult plants in the field (DSr < 20%) in spite of high susceptibility in seedlings against all isolates. This was more frequently identified in *L.c. culinaris*, but also in *L. nigricans* and *L.c. orientalis*. Genetic analysis would be needed to conclude on the inheritance of the identified resistances.

## 5. Conclusions

The fact that hypersensitive, partial and adult plant resistance have been identified within *L.c. culinaris* enables immediate direct use in lentil resistance breeding. Additional valuable sources of resistance have been identified in related species of the primary and secondary gene pools, crossable with cultivated lentils [25,26], making feasible the transfer of rust resistance genes to cultivated lentil. These novel resistance sources should be the base of further studies to establish the genetic, biochemical, and molecular base of rust resistance in lentil. The interest in the incorporation of genetic diversity of wild lentils in pre-breeding and breeding programs is endorsed by recent studies targeting these species [23,57,58,59].

## Figures and Tables

**Table 1:** Description of the sites for field testing. Climatic data correspond to the field seasons only (1 December to 30 June).

Experiment	Season	Site	Latit.	Longit.	Type of Soil	Soil pH	Altit.	Average Tmin (°C)	Average Tmax (°C)	Rain (mm)
Field14	2013–2014	Córdoba, Spain	37°86′ N	4°78′ W	Cambisol	7.8	94	8.3	23.5	342.4
Field15	2014–2015	Córdoba, Spain	37°86′ N	4°78′ W	Cambisol	7.8	94	7.7	23.3	148.8

**Table 2:** Average and range of DS (%) observed at each trial, grouped by Lens species. Field2014 = adult plants in the field 2014; Field2015 = adult plants in the field 2015; ccSPA = seedlings under controlled conditions inoculated with isolate SPA; ccMOR = seedlings under controlled conditions inoculated with isolate MOR; ccFRA = seedlings under controlled conditions inoculated with isolate FRA; ccALG = seedlings under controlled conditions inoculated with isolate ALG; s.d. = standard deviation; ns: not studied.

		*Disease Severity (DS %)*											
	Season/Isolate	*L.c. culinaris*		*L.c. orientalis*		*L.c. odemensis*		*L. ervoides*		*L. lamottei*		*L. nigricans*	
		DS	(Range) s.d.	DS	(Range) s.d.	DS	(Range) s.d.	DS	(Range) s.d.	DS	(Range) s.d.	DS	(Range) s.d.
Adult plants in field trials	Field2014	48	(1–85) 22	ns	ns	23	(12–30) 14	9	(0–20) 12	ns	ns	10	(10–11) 9
	Field2015	25	(0–55) 16	16	(0–40) 13	5	(1–9) 4	9	(0–40) 12	8	(1–15) 11	16	(0–23) 15
Seedlings under controlled conditions	ccSPA	42	(8–73) 13	35	(10–60) 13	31	(23–40) 14	23	(0–40) 13	18	(10–25) 15	25	(5–45) 15
	ccMOR	38	(4–70) 14	28	(0–45) 11	17	(8–18) 13	19	(1–50) 14	16	(15–17) 4	22	(0–40) 10
	ccFRA	43	(0–80) 17	41	(5–70) 15	18	(6–21) 11	24	(3–50) 17	8	(5–10) 3	28	(5–60) 14
	ccALG	32	(0–65) 17	28	(3–50) 14	5	(1–28) 10	16	(0–30) 16	25	(25) -	21	(0–60) 16

**Table 3:** Pearson’s linear correlation coefficient between DS accessed under field and controlled conditions trials. ***, **, * = *p* < 0.001, <0.01, <0.1, respectively; ns = not significant.

	DSfield14	DSfield15	DSccSPA	DSccMOR	DSccFRA
DSfield15	0.46 ***				
DSccSPA	0.09 ns	0.27 ***			
DSccMOR	0.26 ***	0.22 ***	0.43 ***		
DSccFRA	0.24 **	0.19 *	0.36 ***	0.42 ***	
DSccALG	0.10 ns	0.17 *	0.37 ***	0.42 ***	0.21 **

**Table 4:** Number of accessions showing hypersensitive response (IT < 3) across the various *Lens* species against the various isolates of *U. viciae-fabae* ex *L. culinaris*. Seedling tests under controlled conditions.

Isolate	*L.c. culinaris*	*L.c. orientalis*	*L.c. odemensis*	*L. ervoides*	*L. lamottei*	*L. nigricans*
SPA	7 in 510 (1.4%)	1 in 29 (3.4%)	0 in 6 (0%)	2 in 16 (12.5%)	0 in 2 (0%)	1 in 32 (3.1%)
MOR	7 in 445 (1.6%)	0 in30 (0%)	0 in 6 (0%)	3 in 19 (15.8%)	0 in 2 (0%)	2 in 31 (6.4%)
FRA	6 in 373 (1.6%)	1 in 21 (4.8%)	0 in 5 (0%)	1 in 11 (9%)	0 in 2 (0%)	0 in 19 (0%)
ALG	13 in 377 (3.4%)	0 in 26 (0%)	0 in 5 (0%)	3 in 14 (21.4%)	0 in 1 (0%)	2 in 24 (8.3%)

**Table 5:** Selection of accessions carrying hypersensitive response to any of the isolates of *U. viciae-fabae* ex *L. culinaris* studied. CcSPA = seedlings under controlled conditions inoculated with isolate SPA; ccMOR = seedlings under controlled conditions inoculated with isolate MOR; ccFRA = seedlings under controlled conditions inoculated with isolate FRA; ccALG = seedlings under controlled conditions inoculated with isolate ALG; ns: not studied; IT = Infection Type according to Stakman et al. (1962) [29], where IT 0 = no symptoms, IT ; = necrotic flecks, IT 1 = min pustules barely sporulating, IT 2 = necrotic halo surrounding small pustules, IT 3 = chlorotic halo and IT 4 = well-formed pustules with no associated chlorosis or necrosis. Response R (IT < 3), S (IT ≥ 3).

Accession		Species	ccSPA			ccMOR			ccFRA			ccALG		
			IT	DS	Response	IT	DS	Response	IT	DS	Response	IT	DS	Response
1308	ILWL40	*L. ervoides*	1+	6	R	1	3	R	2	3	R	;	0	R
1561	PI518734	*L.c. culinaris*	;	0	R	1	7	R	;	0	R	;	0	R
1168	BGE034194	*L.c. culinaris*	1+	21	R	4	11	S	1+	12	R	1	4	R
1515	PI451763	*L.c. culinaris*	2	38	R	2	28	R	3	45	S	2	10	R
1559	PI518732	*L.c. culinaris*	1	8	R	2	5	R	4	5	S	;	0	R
1599	PI572349	*L. nigricans*	1	5	R	;	0	R	ns	ns	ns	;	0	R
1145	BGE026701	*L.c. culinaris*	3	27	S	1+	12	R	1	7	R	;	0	R
1571	PI572316	*L. ervoides*	;	0	R	1	5	R	ns	ns	ns	ns	ns	ns
1413	PI320944	*L.c. culinaris*	2+	13	R	1	8	R	4	60	S	4	58	S
1632	PI612249	*L.c. orientales*	2+	10	R	4	17	S	2	5	R	4	10	S
1656	BCU001428	*L. nigricans*	4	28	S	1+	15	R	4	5	S	2	5	R
1324	W6 277757	*L.c. culinaris*	3	43	S	3	29	S	4	70	S	1	15	R
1331	W6 27765	*L.c. culinaris*	3	28	S	4	37	S	4	50	S	2	25	R
1165	BGE031070	*L.c. culinaris*	4	40	S	4	17	S	4	23	S	2	4	R
1318	ILWL271	*L. ervoides*	ns	ns	ns	4	10	S	4	47	S	;	0	R
1351	PI209858	*L.c. culinaris*	4	45	S	4	25	S	4	60	S	;	0	R
1361	PI251032	*L.c. culinaris*	3	22	S	3	13	S	4	50	S	1+	18	R
1430	PI345627	*L.c. culinaris*	3	23	S	4	40	S	4	35	S	1+	13	R
1552	PI477921	*L.c. culinaris*	3	37	S	4	15	S	4	20	S	;	0	R
1553	PI486127	*L.c. culinaris*	3	40	S	4	35	S	4	50	S	2	10	R
1586	PI572331	*L. ervoides*	4	20	S	3-	25	S	ns	ns	ns	;	0	R
1288	BGE019580	*L.c. culinaris*	4	24	S	3	9	S	2-	5	R	4	5	S
1470	PI431714	*L.c. culinaris*	4	43	S	4	40	S	2+	30	R	4	40	S
1471	PI431717	*L.c. culinaris*	4	45	S	4	45	S	2+	30	R	4	40	S
1626	PI572396	*L.c. orientalis*	ns	ns	ns	;	0	R	ns	ns	ns	ns	ns	ns

**Table 6:** Summary of responses identified against the four rust isolates studied showing clear isolate-specific responses corresponding to minimum four races, with sources of resistance to each one.

Accessions	Species	Response to Isol SPA	Response to Isol MOR	Response to Isol FRA	Response to Isol ALG
1308, 1561	*L. ervoides*, *L.c. culinaris*	R	R	R	R
1145	*L.c. culinaris*	S	R	R	R
1168	*L.c. culinaris*	R	S	R	R
1515, 1559	*L.c. culinaris*	R	R	S	R
1656	*L. nigricans*	S	R	S	R
1165, 1324, 1331, 1351, 1361, 1430, 1552, 1553	*L.c. culinaris*	S	S	S	R
1632	*L.c. orientales*	R	S	R	S
1288, 1470, 1471	*L.c. culinaris*	S	S	R	S
1413	*L.c. culinaris*	R	R	S	S
Most accessions		S	S	S	S

**Table 7:** Selection of candidates for partial resistance against isolates of *U. viciae-fabae* ex *L. culinaris*. From full data provided in Supplementary Table S1, we highlight here accessions displaying a compatible interaction (IT > 3) but reduced infection at all conditions (DSr < 35%). Data with the same letter per column are not significantly different (LSD test, *p* < 0.01); ns = not studied.

Accession		Species	Adult Plants in the Field		Seedlings Under Controlled Conditions							
					Isol SPA		Isol MOR		Isol FRA		Isol ALG	
			2014 DSr	2015 DSr	IT	DSr	IT	DSr	IT	DSr	IT	DSr
1311	ILWL38	*L. nigricans*	1 b	2 b	4	18 bc	4	9 c	4	24 ab	3	3 d
1303	ILWL31	*L. nigricans*	13 a	ns	4	26 ab	4	27 a	4	19 ab	4	31 a
1658	BCU001430	*L. nigricans*	ns	0.5 c	4	7 d	4	7 c	ns	ns	4	23 ab
1673	BCU001901	*L. nigricans*	ns	10 a	4	21 abc	4	10 bc	ns	ns	ns	ns
1604	PI572356	*L. nigricans*	ns	ns	4	25 ab	4	29 a	4	13 ab	4	12 c
1574	PI572319	*L. ervoides*	ns	0.5 c	4	27 a	3+	29 a	ns	ns	4	31 a
1593	PI572338	*L. ervoides*	ns	ns	4	1 d	ns	ns	4	6 b	4	15 c
1588	PI572333	*L. ervoides*	ns	ns	4	21 abc	4	17 abc	ns	ns	4	31 a
1661	BCU001511	*L. lamottei*	ns	1 bc	4	14 c	4	21 ab	4	6 b	ns	ns
1317	ILWL261	*L.c. odemensis*	33 a	2 b	4	32 a	4	13 bc	4	26 a	3	2 d
1300	BGE34196	*L.c. culinaris*	12 a	ns	4	25 ab	4	6 c	4	10 ab	ns	ns
1446	PI426784	*L.c. culinaris*	ns	ns	4	32 a	3	21 ab	4	10 ab	3	20 bc

**Table 8:** Selection of candidates for adult plant resistance against *U. viciae-fabae* ex *L. culinaris*, susceptible in seedlings (IT > 3, DSr > 50%) but resistant in adult plants in the field (DSr < 20%). Data with the same letter per column are not significantly different (LSD test, *p* < 0.01).

Accession		Species	Field	Seedlings under Controlled Conditions							
				ccSPA		ccMOR		ccFRA		ccALG	
			Field2015 DSr	IT	DSr	IT	DSrIT	IT	DSr	IT	DSr
1660	BCU001510	*L. nigricans*	9 a	4	52 a	4	50 a	ns	ns	4	51 a
1613	PI572374	*L.c. orientalis*	7 b	4	62 a	4	47 a	4	50 a	4	77 a
1455	PI431631	*L.c. culinaris*	2 b	4	68 a	4	69 a	4	88 a	4	69 a
1518	PI468899	*L.c. culinaris*	2 b	4	66 a	4	69 a	4	75 a	4	54 a
1517	PI458503	*L.c. culinaris*	7 b	4	79 a	4	64 a	4	63 a	4	54 a
1519	PI468900	*L.c. culinaris*	7 b	4	68 a	4	61 a	4	63 a	4	69 a
1374	PI297287	*L.c. culinaris*	9 a	4	75 a	4	64 a	4	63 a	4	38 a
1411	PI320940	*L.c. culinaris*	11 a	4	79 a	4	79 a	4	75 a	4	69 a
1565	PI533693	*L.c. culinaris*	15 a	4	78 a	4	71 a	4	88 a	4	69 a
1392	PI299177	*L.c. culinaris*	15 a	4	59 a	4	53 a	4	63 a	4	69 a
1449	PI429369	*L.c. culinaris*	16 a	4	55 a	4	60 a	4	50 a	4	62 a
1501	PI432147	*L.c. culinaris*	18 a	4	73 a	4	64 a	4	63 a	4	77 a
1511	PI432259	*L.c. culinaris*	18 a	4	68 a	4	81 a	4	75 a	4	58 a
1417	PI320953	*L.c. culinaris*	20 a	4	62 a	4	57 a	4	50 a	4	66 a
1387	PI299120	*L.c. culinaris*	22 a	4	86 a	4	71 a	4	50 a	4	69 a
1452	PI431618	*L.c. culinaris*	22 a	4	73 a	4	67 a	4	50 a	4	58 a
1564	PI533691	*L.c. culinaris*	22 a	4	58 a	4	67 a	4	63 a	4	62 a
1473	PI431731	*L.c. culinaris*	24 a	4	59 a	4	50 a	4	63 a	4	69 a
1516	PI451766	*L.c. culinaris*	25 a	4	64 a	4	61 a	4	56 a	4	66 a
1479	PI431809	*L.c. culinaris*	25 a	4	52 a	4	53 a	4	50 a	4	58 a
1403	PI300250	*L.c. culinaris*	25 a	4	51 a	4	50 a	4	50 a	4	62 a

## Data Availability

All relevant data are within the paper and its Supporting Information Files.

## Supplementary Materials

The following supporting information can be downloaded at: https://www.mdpi.com/article/10.3390/plants12030626/s1, Table S1: Response of the *Lens* germplasm accessions to *U. viciae-fabae* ex *L. culinaris* studied. DS = Disease Severity (%); IT = Infection Type according Stakman et al. (1962) [29], 6 where IT 0 = no symptoms, IT ; = necrotic flecks, IT 1 = minute pustules barely sporulating, IT 2 = necrotic halo surrounding small pustules, IT 3 = chlorotic halo and IT 4 = well-formed pustules with 7 no associated chlorosis or necrosis. 8 Ns = not studied
