# Supplementary material for: Identification and Characterization of Resistance to Rust in Lentil and Its Wild Relatives
Source: Plants (Basel). 2023 Jan 31;12(3):626. doi: 10.3390/plants12030626 (PMC9919313; doi:10.3390/plants12030626)
Supplement: Supplementary file 1 [file plants-12-00626-s001.zip › plants-2167345-supplementary.pdf]

plants

MDPI

Identification and characterization of resistance to rust in lentil and its wild relatives

Eleonora Barilli and Diego Rubiales

Supplementary table 1: *Response of the Lens germplasm accessions to U. viciae-fabae ex L. culinaris studied. DS = Disease Severity (%); IT = Infection Type according Stakman et al. (1962) [29], where IT 0 = no symptoms, IT ; = necrotic flecks, IT 1 = min pustules barely sporulating, IT 2 = necrotic halo surrounding small pustules, IT 3 = chlorotic halo and IT 4 = well-formed pustules with no associated chlorosis or necrosis.*
Ns = not studied

Accession code	Donnor's code	species	origin	Field tests Isolate SPA			Seedling tests under controlled conditions							
				2014	2015		Isolate SPA		Isolate MOR		Isolate FRA		Isolate ALG	
				DS(%)	IT	DS(%)	IT	DS(%)	IT	DS(%)	IT	DS(%)	IT	DS(%)
1001	BGE000996	*L.c. culinaris*	Spain	73	4	12	4	30	4	30	4	70	4	13
1002	BGE001017	*L.c. culinaris*	Spain	45	4	15	4	28	4	43	ns	ns	ns	ns
1003	BGE001023	*L.c. culinaris*	Spain	53	4	25	4	33	4	40	4	33	ns	ns
1004	BGE001048	*L.c. culinaris*	Spain	55	4	48	4	27	4	20	4	30	4	13
1005	BGE001055	*L.c. culinaris*	Spain	77	4	30	4	43	4	22	ns	ns	ns	ns
1006	BGE001078	*L.c. culinaris*	Spain	60	ns	ns	4	50	4	18	ns	ns	ns	ns
1007	BGE001080	*L.c. culinaris*	Spain	53	4	43	4	60	4	13	4	13	ns	ns
1008	BGE001087	*L.c. culinaris*	Spain	53	4	25	4	37	4	43	ns	ns	ns	ns
1009	BGE001119	*L.c. culinaris*	Spain	40	4	55	4	47	ns	ns	ns	ns	ns	ns
1010	BGE001140	*L.c. culinaris*	Spain	60	4	23	4	30	ns	ns	ns	ns	ns	ns
1011	BGE001150	*L.c. culinaris*	Spain	60	4	25	4	40	ns	ns	ns	ns	ns	ns
1012	BGE001172	*L.c. culinaris*	Spain	25	4	43	4	47	4	23	4	5	4	17
1013	BGE001378	*L.c. culinaris*	Spain	57	4	16	4	53	4	47	4	28	ns	ns
1014	BGE001394	*L.c. culinaris*	Spain	20	4	25	4	25	4	37	4	13	ns	ns
1015	BGE001398	*L.c. culinaris*	Spain	ns	ns	ns	4	32	ns	ns	ns	ns	ns	ns

1016	BGE001402	*L.c. culinaris*	Spain	30	ns	ns	4	40	ns	ns	ns	ns	ns	ns
1017	BGE001409	*L.c. culinaris*	Spain	40	4	35	4	47	4	50	ns	ns	ns	ns
1018	BGE001415	*L.c. culinaris*	Spain	15	4	15	4	40	4	12	ns	ns	4	2
1019	BGE001430	*L.c. culinaris*	Spain	43	4	35	4	30	4	22	4	12	4	8
1020	BGE001431	*L.c. culinaris*	Spain	50	4	19	4	37	ns	ns	ns	ns	ns	ns
1021	BGE001443	*L.c. culinaris*	Spain	63	4	27	4	38	ns	ns	ns	ns	ns	ns
1022	BGE001459	*L.c. culinaris*	Spain	45	4	27	4	47	ns	ns	ns	ns	ns	ns
1023	BGE001483	*L.c. culinaris*	Spain	60	4	35	4	25	ns	ns	ns	ns	ns	ns
1024	BGE019708	*L.c. culinaris*	Spain	43	4	24	4	43	4	30	ns	ns	4	17
1025	BGE001495	*L.c. culinaris*	Spain	45	4	10	4	57	ns	ns	ns	ns	ns	ns
1026	BGE001499	*L.c. culinaris*	Spain	55	4	28	4	40	4	47	4	50	4	10
1027	BGE001500	*L.c. culinaris*	Spain	52	4	22	4	33	ns	ns	ns	ns	ns	ns
1028	BGE001528	*L.c. culinaris*	Spain	30	ns	14	4	30	4	28	4	15	4	8
1029	BGE008329	*L.c. culinaris*	Spain	60	4	20	4	32	4	33	ns	ns	4	12
1030	BGE001805	*L.c. culinaris*	Spain	40	4	14	4	48	4	43	4	40	4	13
1031	BGE001808	*L.c. culinaris*	Spain	60	4	12	4	40	4	33	ns	ns	ns	ns
1032	BGE001814	*L.c. culinaris*	Spain	47	4	19	4	40	4	40	4	53	4	8
1033	BGE001815	*L.c. culinaris*	Spain	37	4	5	4	40	4	37	4	53	4	23
1034	BGE001821	*L.c. culinaris*	Spain	40	4	35	4	53	ns	ns	ns	ns	ns	ns
1035	BGE001823	*L.c. culinaris*	Spain	65	4	14	4	53	ns	ns	ns	ns	ns	ns
1036	BGE001824	*L.c. culinaris*	Spain	55	4	23	4	35	4	30	4	60	4	20
1037	BGE001833	*L.c. culinaris*	Spain	35	4	40	4	57	4	20	ns	ns	ns	ns
1038	BGE001835	*L.c. culinaris*	Spain	63	4	10	4	60	4	22	ns	ns	ns	ns
1039	BGE001836	*L.c. culinaris*	Spain	47	4	40	4	43	4	53	4	67	4	20
1040	BGE001845	*L.c. culinaris*	Spain	43	4	35	4	50	ns	ns	ns	ns	ns	ns
1041	BGE001846	*L.c. culinaris*	Spain	50	4	35	4	47	ns	ns	ns	ns	ns	ns
1042	BGE001850	*L.c. culinaris*	Spain	60	4	38	4	53	4	37	ns	ns	ns	ns
1043	BGE001851	*L.c. culinaris*	Spain	60	4	33	4	35	4	27	4	70	4	7
1044	BGE001853	*L.c. culinaris*	Spain	47	4	25	4	63	4	47	ns	ns	ns	ns
1045	BGE001854	*L.c. culinaris*	Spain	50	4	28	4	67	4	53	4	57	4	43
1046	BGE001862	*L.c. culinaris*	Spain	37	4	25	4	47	4	53	4	18	4	30
1047	BGE001867	*L.c. culinaris*	Spain	57	4	15	4	60	4	37	4	40	4	33
1048	BGE001868	*L.c. culinaris*	Spain	50	4	40	4	40	4	50	ns	ns	ns	ns
1049	BGE001875	*L.c. culinaris*	Spain	67	4	28	4	60	4	25	ns	ns	ns	ns
1050	BGE001879	*L.c. culinaris*	Spain	75	4	50	4	28	ns	ns	ns	ns	ns	ns
1051	BGE001880	*L.c. culinaris*	Spain	47	4	28	4	30	4	33	4	18	4	5
1052	BGE001884	*L.c. culinaris*	Spain	50	4	35	4	47	4	28	4	50	4	10
1053	BGE001890	*L.c. culinaris*	Spain	55	4	28	4	50	ns	ns	ns	ns	ns	ns
1054	BGE001899	*L.c. culinaris*	Spain	40	4	23	4	57	4	18	ns	ns	ns	ns

1055	BGE001908	*L.c. culinaris*	Spain	43	4	43	4	47	ns	ns	ns	ns	ns	ns
1056	BGE001914	*L.c. culinaris*	Spain	53	4	19	4	37	4	28	ns	ns	ns	ns
1057	BGE001920	*L.c. culinaris*	Spain	45	4	27	4	25	ns	ns	ns	ns	ns	ns
1058	BGE001924	*L.c. culinaris*	Spain	33	4	25	4	37	4	20	ns	ns	ns	ns
1059	BGE002058	*L.c. culinaris*	Spain	70	4	30	4	40	4	22	4	22	4	37
1060	BGE4243	*L.c. culinaris*	Spain	60	4	40	4	33	ns	ns	ns	ns	ns	ns
1061	BGE004244	*L.c. culinaris*	Spain	40	4	32	4	43	ns	ns	ns	ns	ns	ns
1062	BGE004245	*L.c. culinaris*	Spain	63	4	50	4	23	ns	ns	ns	ns	ns	ns
1063	BGE004246	*L.c. culinaris*	Spain	63	4	15	4	22	ns	ns	ns	ns	ns	ns
1064	BGE004247	*L.c. culinaris*	Spain	63	4	35	4	27	ns	ns	ns	ns	ns	ns
1065	BGE004248	*L.c. culinaris*	Spain	55	4	20	4	47	4	38	4	25	4	50
1066	BGE004249	*L.c. culinaris*	Spain	30	4	15	4	57	ns	ns	ns	ns	ns	ns
1067	BGE004250	*L.c. culinaris*	Spain	53	4	15	4	15	4	32	4	20	4	8
1068	BGE004251	*L.c. culinaris*	Spain	37	4	11	4	18	ns	ns	ns	ns	ns	ns
1069	BGE004252	*L.c. culinaris*	Spain	15	4	7	4	30	4	43	4	40	4	47
1070	BGE004420	*L.c. culinaris*	Spain	65	4	35	4	53	ns	ns	ns	ns	ns	ns
1071	BGE008329	*L.c. culinaris*	Spain	60	4	28	4	43	4	50	4	50	4	17
1072	BGE008688	*L.c. culinaris*	Spain	50	4	9	4	37	ns	ns	ns	ns	ns	ns
1073	BGE08690	*L.c. culinaris*	Spain	55	4	27	4	57	ns	ns	ns	ns	ns	ns
1074	BGE008691	*L.c. culinaris*	Spain	47	4	33	4	50	ns	ns	ns	ns	ns	ns
1075	BGE008692	*L.c. culinaris*	Spain	45	4	43	4	50	ns	ns	ns	ns	ns	ns
1076	BGE008693	*L.c. culinaris*	Spain	40	4	15	4	22	4	18	4	30	4	8
1077	BGE008694	*L.c. culinaris*	Spain	47	4	32	4	25	4	47	4	27	4	27
1078	BGE8695	*L.c. culinaris*	Spain	35	4	3	4	23	4	12	ns	ns	ns	ns
1079	BGE008696	*L.c. culinaris*	Spain	45	4	6	4	25	4	35	ns	ns	ns	ns
1080	BGE008697	*L.c. culinaris*	Spain	55	4	18	4	18	4	40	4	57	4	18
1081	BGE008698	*L.c. culinaris*	Spain	53	4	18	4	22	4	23	ns	ns	ns	ns
1082	BGE008700	*L.c. culinaris*	Spain	53	4	28	4	30	4	35	ns	ns	ns	ns
1083	BGE08701	*L.c. culinaris*	Spain	80	4	40	4	30	4	40	ns	ns	ns	ns
1084	BGE008726	*L.c. culinaris*	Spain	47	4	12	4	40	4	50	ns	ns	ns	ns
1085	BGE008979	*L.c. culinaris*	Spain	26	4	23	4	28	4	37	ns	ns	ns	ns
1086	BGE008991	*L.c. culinaris*	Spain	35	4	43	4	47	ns	ns	ns	ns	ns	ns
1087	BGE089982	*L.c. culinaris*	Spain	45	4	37	4	20	ns	ns	ns	ns	ns	ns
1088	BGE008986	*L.c. culinaris*	Spain	30	4	23	4	40	4	43	4	43	4	12
1089	BGE011074	*L.c. culinaris*	Spain	75	4	35	4	37	4	53	4	63	4	12
1090	BGE011075	*L.c. culinaris*	Spain	53	4	33	3	33	4	47	4	63	4	23
1091	BGE011077	*L.c. culinaris*	Spain	50	4	20	4	30	4	28	4	8	4	4
1092	BGE011078	*L.c. culinaris*	Spain	80	4	15	4	63	ns	ns	ns	ns	ns	ns
1093	BGE011082	*L.c. culinaris*	Spain	47	4	19	4	25	ns	ns	ns	ns	ns	ns

1094	BGE011085	*L.c. culinaris*	Spain	47	4	32	4	27	ns	ns	ns	ns	ns	ns
1095	BGE011086	*L.c. culinaris*	Spain	60	4	28	4	37	4	23	4	25	4	2
1096	BGE011087	*L.c. culinaris*	Spain	40	4	23	4	30	ns	ns	ns	ns	ns	ns
1097	BGE011088	*L.c. culinaris*	Spain	15	4	20	4	50	ns	ns	ns	ns	ns	ns
1098	BGE011089	*L.c. culinaris*	Spain	20	4	19	4	57	4	67	4	60	4	17
1099	BGE011091	*L.c. culinaris*	Spain	55	4	28	4	37	ns	ns	ns	ns	ns	ns
1100	BGE011092	*L.c. culinaris*	Spain	70	4	38	4	28	4	53	4	70	4	4
1101	BGE011094	*L.c. culinaris*	Spain	37	4	45	4	32	ns	ns	ns	ns	ns	ns
1102	BGE011095	*L.c. culinaris*	Spain	63	4	15	4	33	4	50	ns	ns	ns	ns
1103	BGE011096	*L.c. culinaris*	Spain	50	4	33	4	37	4	50	ns	ns	ns	ns
1104	BGE011085	*L.c. culinaris*	Spain	60	4	29	4	20	4	28	ns	ns	ns	ns
1105	BGE014000	*L.c. culinaris*	Spain	60	4	30	4	60	4	50	ns	ns	ns	ns
1106	BGE016344	*L.c. culinaris*	Spain	60	4	24	4	47	4	18	ns	ns	ns	ns
1107	BGE016345	*L.c. culinaris*	Spain	63	4	40	4	57	4	30	ns	ns	ns	ns
1108	BGE016346	*L.c. culinaris*	Spain	67	4	33	4	43	4	28	ns	ns	ns	ns
1109	BGE016348	*L.c. culinaris*	Spain	53	4	47	4	43	4	30	4	67	4	17
1110	BGE016350	*L.c. culinaris*	Spain	37	4	14	4	57	4	37	4	43	ns	ns
1111	BGE016352	*L.c. culinaris*	Spain	60	4	33	4	50	4	32	4	20	4	10
1112	BGE016355	*L.c. culinaris*	Spain	40	4	15	4	53	4	35	4	27	4	13
1113	BGE016356	*L.c. culinaris*	Spain	57	4	30	4	43	4	43	ns	ns	ns	ns
1114	BGE016357	*L.c. culinaris*	Spain	50	4	30	4	60	ns	ns	ns	ns	ns	ns
1115	BGE016358	*L.c. culinaris*	Spain	50	4	30	4	50	4	63	ns	ns	4	37
1116	BGE016359	*L.c. culinaris*	Spain	40	4	20	4	53	4	57	4	47	4	33
1117	BGE016362	*L.c. culinaris*	Spain	83	4	25	4	50	4	70	ns	ns	ns	ns
1118	BGE016363	*L.c. culinaris*	Spain	85	4	30	4	23	4	47	ns	ns	ns	ns
1119	BGE016365	*L.c. culinaris*	Spain	75	4	35	4	37	4	37	ns	ns	ns	ns
1120	BGE019696	*L.c. culinaris*	Spain	40	4	16	4	55	4	50	4	37	4	7
1121	BGE019698	*L.c. culinaris*	Spain	63	4	28	4	40	4	60	4	43	4	12
1122	BGE019699	*L.c. culinaris*	Spain	35	4	12	4	30	ns	ns	ns	ns	ns	ns
1123	BGE019701	*L.c. culinaris*	Spain	50	4	21	4	27	4	27	4	38	4	1
1124	BGE019708	*L.c. culinaris*	Spain	53	4	33	4	50	4	43	4	43	4	17
1125	BGE019710	*L.c. culinaris*	Spain	55	4	33	4	47	ns	ns	ns	ns	ns	ns
1126	BGE019716	*L.c. culinaris*	Spain	50	4	37	4	27	4	47	ns	ns	ns	ns
1127	BGE019721	*L.c. culinaris*	Spain	47	4	28	4	63	4	45	ns	ns	ns	ns
1128	BGE019729	*L.c. culinaris*	Spain	60	4	22	4	47	4	63	4	32	4	8
1129	BGE022153	*L.c. culinaris*	Spain	35	4	10	4	57	4	47	4	53	4	27
1130	BGE022526	*L.c. culinaris*	Spain	40	4	13	4	37	4	53	4	53	4	20
1131	BGE023248	*L.c. culinaris*	Spain	13	4	4	4	60	4	40	4	45	4	17
1132	BGE023249	*L.c. culinaris*	Spain	47	4	28	4	50	ns	ns	ns	ns	ns	ns

1133	BGE023250	*L.c. culinaris*	Spain	35	4	35	4	57	4	43	4	32	4	27
1134	BGE023655	*L.c. culinaris*	Spain	30	4	30	4	50	ns	ns	ns	ns	ns	ns
1135	BGE024338	*L.c. culinaris*	Spain	53	4	20	4	63	ns	ns	ns	ns	ns	ns
1136	BGE024339	*L.c. culinaris*	Spain	33	4	8	4	40	ns	ns	ns	ns	ns	ns
1137	BGE025292	*L.c. culinaris*	Spain	1	4	7	4	30	4	38	4	32	4	8
1138	BGE025596	*L.c. culinaris*	Spain	55	4	5	4	37	4	37	4	47	ns	ns
1139	BGE025598	*L.c. culinaris*	Spain	40	4	19	4	30	4	22	4	32	4	17
1140	BGE025599	*L.c. culinaris*	Spain	20	4	30	4	57	4	43	4	47	4	12
1141	BGE025600	*L.c. culinaris*	Spain	50	4	25	4	40	4	47	4	27	4	22
1142	BGE025602	*L.c. culinaris*	Spain	50	4	35	4	33	ns	ns	ns	ns	ns	ns
1143	BGE025718	*L.c. culinaris*	Spain	40	4	25	4	57	ns	ns	ns	ns	ns	ns
1144	BGE025720	*L.c. culinaris*	Spain	44	4	22	4	37	4	8	4	67	4	10
1145	BGE026701	*L.c. culinaris*	Spain	3	4	9	3	23	1+	12	1	7	;	0
1146	BGE027126	*L.c. culinaris*	Spain	50	4	38	4	37	4	27	4	63	4	18
1147	BGE027127	*L.c. culinaris*	Spain	70	4	13	4	37	4	43	4	57	ns	ns
1148	BGE029091	*L.c. culinaris*	Spain	45	4	18	4	47	4	30	ns	ns	ns	ns
1149	BGE031050	*L.c. culinaris*	Spain	47	4	50	4	28	4	43	ns	ns	ns	ns
1150	BGE031051	*L.c. culinaris*	Spain	43	4	38	4	37	4	33	ns	ns	ns	ns
1151	BGE031052	*L.c. culinaris*	Spain	40	4	17	4	30	4	57	ns	ns	ns	ns
1152	BGE031054	*L.c. culinaris*	Spain	65	4	37	4	40	4	67	ns	ns	ns	ns
1153	BGE031055	*L.c. culinaris*	Spain	60	ns	ns	4	37	4	50	ns	ns	ns	ns
1154	BGE031056	*L.c. culinaris*	Spain	30	4	30	4	40	ns	ns	ns	ns	ns	ns
1155	BGE031059	*L.c. culinaris*	Spain	40	4	40	4	43	ns	ns	ns	ns	ns	ns
1156	BGE031058	*L.c. culinaris*	Spain	53	4	30	4	47	4	27	4	35	4	10
1157	BGE031060	*L.c. culinaris*	Spain	80	4	32	4	43	4	32	4	23	4	1
1158	BGE031061	*L.c. culinaris*	Spain	50	4	43	4	47	4	27	4	17	4	20
1159	BGE031062	*L.c. culinaris*	Spain	60	4	30	4	60	ns	ns	ns	ns	ns	ns
1160	BGE031063	*L.c. culinaris*	Spain	67	4	35	4	57	4	57	4	53	4	12
1161	BGE031064	*L.c. culinaris*	Spain	55	4	25	4	50	4	67	4	63	ns	ns
1162	BGE031065	*L.c. culinaris*	Spain	45	4	20	4	45	4	48	4	60	ns	ns
1163	BGE031068	*L.c. culinaris*	Spain	30	4	23	4	37	4	60	4	63	ns	ns
1164	BGE031069	*L.c. culinaris*	Spain	50	4	35	4	33	4	40	4	18	ns	ns
1165	BGE031070	*L.c. culinaris*	Spain	53	4	27	4	40	4	17	4	23	2	4
1166	BGE032283	*L.c. culinaris*	Spain	23	4	19	4	37	4	40	4	42	4	5
1167	BGE032289	*L.c. culinaris*	Spain	65	4	45	4	37	ns	ns	ns	ns	ns	ns
1168	BGE034194	*L.c. culinaris*	Spain	1	;(2)	9	1+	21	4	11	1+	12	1	4
1170	BGE001861	*L.c. culinaris*	Spain	40	4	35	4	63	4	60	ns	ns	ns	ns
1171	BGE008983	*L.c. culinaris*	Spain	23	4	18	4	57	4	40	ns	ns	ns	ns

1172	BGE037770	*L.c. culinaris*	Spain	47	4	19	4	40	ns	ns	ns	ns	ns	ns
1173	BGE039490	*L.c. culinaris*	Spain	65	4	45	4	40	ns	ns	ns	ns	ns	ns
1174	BGE039491	*L.c. culinaris*	Spain	50	4	40	4	43	ns	ns	ns	ns	ns	ns
1175	BGE016347	*L.c. culinaris*	Spain	45	4	30	4	33	4	33	4	30	4	43
1176	BGE016353	*L.c. culinaris*	Spain	47	4	35	4	43	4	37	4	18	4	43
1177	BGE016354	*L.c. culinaris*	Spain	37	4	27	4	60	4	57	4	47	4	40
1178	BGE016360	*L.c. culinaris*	Spain	30	4	15	4	63	ns	ns	ns	ns	ns	ns
1179	BGE016364	*L.c. culinaris*	Spain	40	4	38	4	40	ns	ns	ns	ns	ns	ns
1180	BGE019700	*L.c. culinaris*	Spain	50	4	37	4	37	4	40	4	50	4	23
1181	BGE025719	*L.c. culinaris*	Spain	30	4	20	4	43	4	57	ns	ns	ns	ns
1182	BGE029684	*L.c. culinaris*	Spain	30	4	14	4	37	ns	ns	ns	ns	ns	ns
1183	BGE029685	*L.c. culinaris*	Spain	23	4	50	4	47	4	47	ns	ns	ns	ns
1184	BGE031048	*L.c. culinaris*	Spain	3	4	7	4	47	4	27	ns	ns	ns	ns
1185	BGE032284	*L.c. culinaris*	Spain	57	4	30	4	70	4	43	4	77	4	17
1186	BGE032290	*L.c. culinaris*	Spain	50	4	33	4	70	4	50	4	73	4	37
1187	BGE034195	*L.c. culinaris*	Spain	50	4	40	4	43	ns	ns	ns	ns	ns	ns
1188	BGE034196	*L.c. culinaris*	Spain	20	4	22	4	43	ns	ns	ns	ns	ns	ns
1189	BGE034197	*L.c. culinaris*	Spain	40	4	30	4	28	ns	ns	ns	ns	ns	ns
1190	BGE034413	*L.c. culinaris*	Spain	70	4	45	4	42	4	42	4	25	4	7
1191	BGE040543	*L.c. culinaris*	Spain	60	4	38	4	33	4	30	4	33	4	8
1192	BGE040544	*L.c. culinaris*	Spain	30	4	28	4	37	4	40	4	35	4	4
1193	BGE040545	*L.c. culinaris*	Spain	47	4	20	4	33	4	25	4	15	4	5
1194	BGE040546	*L.c. culinaris*	Spain	43	4	10	4	20	4	15	4	20	4	2
1195	BGE040547	*L.c. culinaris*	Spain	40	4	4	4	25	4	40	4	22	4	13
1196	BGE040548	*L.c. culinaris*	Spain	50	4	33	4	25	4	30	4	33	4	17
1197	BGE040549	*L.c. culinaris*	Spain	35	4	38	4	30	4	47	4	25	4	13
1283	BGE001882	*L.c. culinaris*	Spain	80	4	27	ns	ns	ns	ns	4	50	4	23
1284	BGE001885	*L.c. culinaris*	Spain	60	4	25	ns	ns	ns	ns	4	40	4	20
1285	BGE001892	*L.c. culinaris*	Spain	67	4	40	ns	ns	ns	ns	4	57	4	30
1286	BGE016366	*L.c. culinaris*	Spain	35	4	20	ns	ns	ns	ns	4	60	ns	ns
1288	BGE019580	*L.c. culinaris*		30	ns	ns	4	24	3	9	2-	5	4	5
1289	BGE019582	*L.c. culinaris*		57	4	38	ns	ns	ns	ns	4	35	4	5
1292	BGE024343	*L.c. culinaris*	Spain	57	4	11	4	45	ns	ns	4	50	4	7
1293	BGE024693	*L.c. culinaris*	Spain	77	4	45	ns	ns	ns	ns	4	70	ns	ns
1294	BGE029092	*L.c. culinaris*	Spain	60	4	33	4	15	ns	ns	4	17	4	2
1295	BGE031066	*L.c. culinaris*	Spain	30	ns	ns	ns	ns	ns	ns	4	40	4	10
1296	BGE031067	*L.c. culinaris*	Spain	50	4	27	ns	ns	ns	ns	4	63	4	7
1297	BGE032286	*L.c. culinaris*	Spain	40	4	37	ns	ns	ns	ns	4	50	4	23

1298	BGE032290	*L.c. culinaris*	Spain	57	4	45	4	43	ns	ns	4	33	ns	ns
1299	BGE132291	*L.c. culinaris*	Spain	60	4	40	4	20	ns	ns	4	40	ns	ns
1300	BGE034196	*L.c. culinaris*	Spain	10	ns	ns	4	18	4	4	4	8	ns	ns
1322	W6 27754	*L.c. culinaris*	USA	ns	4	40	4	48	4	48	4	30	4	45
1323	W6 27756	*L.c. culinaris*	USA	ns	ns	ns	4	42	4	27	4	40	4	38
1324	W6 27757	*L.c. culinaris*	USA	ns	ns	ns	3	43	3	29	4	70	1	15
1325	W6 27758	*L.c. culinaris*	USA	ns	ns	ns	4	42	4	40	4	50	4	50
1326	W6 27759	*L.c. culinaris*	USA	ns	ns	ns	4	50	4	37	4	60	4	50
1327	W6 27760	*L.c. culinaris*	USA	ns	ns	ns	4	53	4	30	4	40	4	50
1328	W6 27762	*L.c. culinaris*	USA	ns	ns	ns	4	45	4	37	4	50	4	55
1329	W6 27763	*L.c. culinaris*	USA	ns	ns	ns	4	47	4	40	4	40	4	45
1330	W6 27764	*L.c. culinaris*	USA	ns	ns	ns	4	33	4	32	4	30	4	28
1331	W6 27765	*L.c. culinaris*	USA	ns	ns	ns	3	28	4	37	4	50	2	25
1332	W6 27766	*L.c. culinaris*	USA	ns	ns	ns	4	55	4	38	4	70	4	45
1333	W6 27767	*L.c. culinaris*	USA	ns	ns	ns	4	42	4	43	4	40	4	35
1334	W6 27780	*L.c. culinaris*	USA	ns	ns	ns	4	43	4	48	4	50	4	45
1335	W6 27781	*L.c. culinaris*	USA	ns	ns	ns	4	52	4	42	4	60	4	50
1336	W6 27782	*L.c. culinaris*	USA	ns	ns	ns	4	52	4	48	4	70	4	45
1338	PI169534	*L.c. culinaris*	USA	ns	4	25	4	45	4	28	4	50	4	40
1339	PI175754	*L.c. culinaris*	USA	ns	4	30	4	57	4	43	4	60	4	40
1340	PI177430	*L.c. culinaris*	USA	ns	4	30	4	57	4	43	4	60	4	50
1341	PI178939	*L.c. culinaris*	USA	ns	4	3	4	23	4	27	4	40	4	35
1342	PI178940	*L.c. culinaris*	USA	ns	4	20	4	47	4	45	4	50	4	43
1343	PI178971	*L.c. culinaris*	USA	ns	4	40	4	43	4	40	4	50	4	33
1344	PI179324	*L.c. culinaris*	USA	ns	4	27	4	43	4	30	4	40	4	45
1345	PI181771	*L.c. culinaris*	USA	ns	4	40	4	36	4	43	4	50	4	40
1346	PI182217	*L.c. culinaris*	USA	ns	4	40	4	43	4	45	4	60	4	30
1347	PI193547	*L.c. culinaris*	USA	ns	4	25	4	33	4	37	4	50	4	48
1348	PI193548	*L.c. culinaris*	USA	ns	4	55	4	47	4	35	4	50	4	45
1349	PI193817	*L.c. culinaris*	USA	ns	4	40	4	33	4	33	4	40	4	20
1350	PI207492	*L.c. culinaris*	USA	ns	4	18	4	37	4	33	4	40	4	30
1351	PI209858	*L.c. culinaris*	USA	ns	4	1	4	45	4	25	4	60	;	0
1352	PI211052	*L.c. culinaris*	USA	ns	4	45	4	35	4	42	4	40	4	28
1353	PI211602	*L.c. culinaris*	USA	ns	4	25	4	47	4	48	4	60	4	30
1354	PI212100	*L.c. culinaris*	USA	ns	4	28	4	43	4	30	4	40	4	35
1355	PI212610	*L.c. culinaris*	USA	ns	4	35	4	40	4	35	3	25	4	40
1356	PI217949	*L.c. culinaris*	USA	ns	4	25	4	23	4	13	ns	ns	4	50
1358	PI238758	*L.c. culinaris*	USA	ns	4	30	4	48	4	48	4	50	4	50

1359	PI250156	*L.c. culinaris*	USA	ns	4	40	4	37	4	33	4	40	4	43
1360	PI250158	*L.c. culinaris*	USA	ns	4	3	4	30	4	33	4	40	4	38
1361	PI251032	*L.c. culinaris*	USA	ns	4	6	3	22	3	13	4	50	1+	18
1362	PI251248	*L.c. culinaris*	USA	ns	ns	ns	4	38	4	28	4	40	4	45
1366	PI273664	*L.c. culinaris*	USA	ns	4	40	4	47	4	43	4	40	4	35
1367	PI283604	*L.c. culinaris*	USA	ns	4	20	4	60	4	55	4	40	4	53
1368	PI289070	*L.c. culinaris*	USA	ns	4	25	4	73	4	55	4	40	4	50
1369	PI289073	*L.c. culinaris*	USA	ns	4	19	4	53	4	37	4	50	4	45
1370	PI289079	*L.c. culinaris*	USA	ns	4	8	4	43	4	32	4	40	4	35
1371	PI290716	*L.c. culinaris*	USA	ns	4	17	4	27	4	32	3	20	4	30
1372	PI297284	*L.c. culinaris*	USA	ns	4	15	4	43	4	40	4	45	4	50
1373	PI297285	*L.c. culinaris*	USA	ns	4	15	4	50	4	38	4	50	4	48
1374	PI297287	*L.c. culinaris*	USA	ns	4	5	4	55	4	45	4	50	4	25
1375	PI297754	*L.c. culinaris*	USA	ns	4	19	4	43	4	38	4	50	4	55
1376	PI297772	*L.c. culinaris*	USA	ns	4	21	4	47	4	37	4	40	4	45
1377	PI297787	*L.c. culinaris*	USA	ns	4	20	4	40	4	25	4	40	4	30
1378	PI298023	*L.c. culinaris*	USA	ns	4	48	4	60	4	40	4	45	4	25
1379	PI298121	*L.c. culinaris*	USA	ns	4	25	4	48	4	27	4	25	4	35
1380	PI298122	*L.c. culinaris*	USA	ns	4	24	4	43	4	38	4	20	4	40
1381	PI298357	*L.c. culinaris*	USA	ns	4	35	4	53	4	42	4	45	4	30
1382	PI298631	*L.c. culinaris*	USA	ns	ns	ns	4	47	4	65	4	50	4	45
1383	PI298644	*L.c. culinaris*	USA	ns	4	45	4	53	4	52	4	45	4	10
1384	PI298922	*L.c. culinaris*	USA	ns	4	5	4	52	4	33	4	45	4	40
1385	PI298923	*L.c. culinaris*	USA	ns	4	50	4	57	4	33	4	35	4	45
1386	PI299116	*L.c. culinaris*	USA	ns	4	38	4	43	4	37	4	40	4	48
1387	PI299120	*L.c. culinaris*	USA	ns	4	12	4	63	4	50	4	40	4	45
1388	PI299121	*L.c. culinaris*	USA	ns	4	27	4	55	4	45	4	50	4	50
1389	PI299126	*L.c. culinaris*	USA	ns	ns	ns	4	52	4	48	4	50	4	50
1391	PI299164	*L.c. culinaris*	USA	ns	4	20	4	43	4	40	4	40	4	65
1392	PI299177	*L.c. culinaris*	USA	ns	4	8	4	43	4	37	4	50	4	45
1394	PI299215	*L.c. culinaris*	USA	ns	4	25	4	35	4	45	4	50	4	48
1395	PI299237	*L.c. culinaris*	USA	ns	4	33	4	38	4	42	4	60	4	40
1398	PI299351	*L.c. culinaris*	USA	ns	4	22	4	57	4	37	4	30	4	30
1400	PI299366	*L.c. culinaris*	USA	ns	4	33	4	47	4	33	4	50	4	53
1401	PI299371	*L.c. culinaris*	USA	ns	4	38	4	52	4	40	4	60	4	50
1403	PI300250	*L.c. culinaris*	USA	ns	4	14	4	37	4	35	4	40	4	40
1404	PI302398	*L.c. culinaris*	USA	ns	ns	ns	4	42	4	38	4	40	4	45
1405	PI308614	*L.c. culinaris*	USA	ns	4	8	4	42	4	43	4	40	4	25
1406	PI311107	*L.c. culinaris*	USA	ns	4	33	4	50	4	60	4	50	4	40

1407	PI312175	*L.c. culinaris*	USA	ns	4	35	4	35	4	40	4	40	4	40
1408	PI320935	*L.c. culinaris*	USA	ns	4	33	4	43	4	28	ns	ns	4	40
1409	PI320936	*L.c. culinaris*	USA	ns	4	10	4	43	4	19	4	15	4	35
1410	PI320937	*L.c. culinaris*	USA	ns	4	15	4	40	4	40	ns	ns	4	45
1411	PI320940	*L.c. culinaris*	USA	ns	4	6	4	58	4	55	4	60	4	45
1412	PI320941	*L.c. culinaris*	USA	ns	4	42	4	30	4	38	4	40	4	28
1413	PI320944	*L.c. culinaris*	USA	ns	4	30	2+	13	1	8	4	60	4	58
1414	PI320945	*L.c. culinaris*	USA	ns	4	8	4	47	4	37	4	50	4	28
1415	PI320946	*L.c. culinaris*	USA	ns	4	3	4	35	4	43	4	40	4	50
1416	PI320952	*L.c. culinaris*	USA	ns	ns	ns	4	45	4	43	4	30	4	25
1417	PI320953	*L.c. culinaris*	USA	ns	4	11	4	45	4	40	4	40	4	43
1418	PI320954	*L.c. culinaris*	USA	ns	4	45	4	48	4	38	4	50	4	43
1419	PI329157	*L.c. culinaris*	USA	ns	4	15	4	43	4	30	4	60	4	40
1423	PI339283	*L.c. culinaris*	USA	ns	4	25	4	47	4	38	4	40	4	58
1424	PI339285	*L.c. culinaris*	USA	ns	4	23	4	48	4	42	4	60	4	38
1426	PI339292	*L.c. culinaris*	USA	ns	4	30	4	43	4	30	4	35	4	25
1427	PI339310	*L.c. culinaris*	USA	ns	4	28	4	48	4	42	4	40	4	45
1428	PI339318	*L.c. culinaris*	USA	ns	4	30	4	45	4	28	4	25	4	23
1429	PI343026	*L.c. culinaris*	USA	ns	4	35	4	42	4	30	4	45	4	30
1430	PI345627	*L.c. culinaris*	USA	ns	4	1	3	23	4	40	4	35	1+	13
1431	PI357225	*L.c. culinaris*	USA	ns	4	6	4	42	4	32	4	45	4	45
1432	PI358602	*L.c. culinaris*	USA	ns	4	8	4	47	4	38	4	50	4	30
1433	PI368647	*L.c. culinaris*	USA	ns	4	2	4	40	4	42	4	35	4	40
1434	PI368651	*L.c. culinaris*	USA	ns	ns	ns	4	50	4	38	4	45	4	38
1435	PI370481	*L.c. culinaris*	USA	ns	ns	ns	4	48	4	32	4	70	4	58
1436	PI374116	*L.c. culinaris*	USA	ns	4	25	4	35	4	30	4	35	4	45
1437	PI374117	*L.c. culinaris*	USA	ns	4	27	3	35	4	35	4	70	3	23
1438	PI374120	*L.c. culinaris*	USA	ns	ns	ns	4	42	4	43	4	25	4	30
1439	PI379368	*L.c. culinaris*	USA	ns	4	33	4	40	4	40	4	40	4	45
1440	PI383682	*L.c. culinaris*	USA	ns	4	35	4	47	4	48	4	40	4	38
1443	PI420929	*L.c. culinaris*	USA	ns	4	30	4	40	4	42	4	60	4	43
1444	PI426202	*L.c. culinaris*	USA	ns	4	35	4	38	4	37	4	30	4	38
1445	PI426778	*L.c. culinaris*	USA	ns	4	17	4	42	4	43	4	50	4	38
1446	PI426784	*L.c. culinaris*	USA	ns	ns	ns	4	23	3	15	4	8	3	13
1447	PI426797	*L.c. culinaris*	USA	ns	4	1	4	35	4	35	4	30	4	43
1448	PI426807	*L.c. culinaris*	USA	ns	4	16	4	35	4	42	4	15	4	48
1449	PI429369	*L.c. culinaris*	USA	ns	4	9	4	40	4	42	4	40	4	40
1450	PI429838	*L.c. culinaris*	USA	ns	4	40	4	48	4	42	4	40	4	30
1452	PI431618	*L.c. culinaris*	USA	ns	4	12	4	53	4	47	4	40	4	38

1453	PI431622	*L.c. culinaris*	USA	ns	4	33	4	30	4	43	4	40	4	48
1454	PI431630	*L.c. culinaris*	USA	ns	4	35	4	32	4	40	4	50	4	45
1455	PI431631	*L.c. culinaris*	USA	ns	4	1	4	50	4	48	4	70	4	45
1456	PI431633	*L.c. culinaris*	USA	ns	4	24	4	50	4	38	4	60	4	45
1457	PI431636	*L.c. culinaris*	USA	ns	4	22	4	50	4	45	4	70	4	45
1458	PI431640	*L.c. culinaris*	USA	ns	ns	40	4	45	4	38	4	50	4	48
1459	PI431642	*L.c. culinaris*	USA	ns	4	30	4	50	4	38	3	30	4	45
1460	PI431643	*L.c. culinaris*	USA	ns	4	40	3	33	4	22	4	50	4	50
1461	PI431656	*L.c. culinaris*	USA	ns	4	35	4	50	4	43	4	50	4	45
1462	PI431662	*L.c. culinaris*	USA	ns	4	45	4	40	4	43	4	40	4	40
1463	PI431663	*L.c. culinaris*	USA	ns	4	40	4	42	4	43	4	60	4	40
1464	PI431666	*L.c. culinaris*	USA	ns	4	33	4	40	4	48	4	40	4	38
1465	PI431675	*L.c. culinaris*	USA	ns	4	38	4	40	4	47	4	40	4	45
1466	PI431679	*L.c. culinaris*	USA	ns	4	30	4	50	4	37	4	30	4	38
1467	PI431684	*L.c. culinaris*	USA	ns	4	20	4	50	4	37	4	40	4	40
1468	PI431705	*L.c. culinaris*	USA	ns	4	40	4	43	4	32	4	40	4	38
1469	PI431710	*L.c. culinaris*	USA	ns	4	25	4	47	4	40	4	50	4	40
1470	PI431714	*L.c. culinaris*	USA	ns	4	18	4	43	4	40	2+	30	4	40
1471	PI431717	*L.c. culinaris*	USA	ns	4	30	4	45	4	45	2+	30	4	40
1472	PI431728	*L.c. culinaris*	USA	ns	4	19	4	45	4	45	4	30	4	35
1473	PI431731	*L.c. culinaris*	USA	ns	4	13	4	43	4	35	4	50	4	45
1474	PI431739	*L.c. culinaris*	USA	ns	4	40	4	43	4	43	4	60	4	50
1475	PI431753	*L.c. culinaris*	USA	ns	4	25	4	47	4	42	4	60	4	45
1476	PI431756	*L.c. culinaris*	USA	ns	4	27	4	48	4	32	4	60	4	43
1477	PI431768	*L.c. culinaris*	USA	ns	4	30	4	55	4	37	4	80	4	50
1478	PI431774	*L.c. culinaris*	USA	ns	4	20	4	52	4	40	4	45	4	43
1479	PI431809	*L.c. culinaris*	USA	ns	4	14	4	38	4	37	4	40	4	38
1480	PI431810	*L.c. culinaris*	USA	ns	4	42	4	53	4	32	4	40	4	35
1481	PI431824	*L.c. culinaris*	USA	ns	4	28	4	43	4	53	4	40	4	45
1482	PI431863	*L.c. culinaris*	USA	ns	ns	ns	4	52	4	45	4	40	4	50
1483	PI431873	*L.c. culinaris*	USA	ns	4	35	4	40	4	32	4	50	4	40
1484	PI431884	*L.c. culinaris*	USA	ns	4	40	4	43	4	35	4	60	4	55
1485	PI431888	*L.c. culinaris*	USA	ns	4	28	4	42	4	37	4	50	4	48
1486	PI431893	*L.c. culinaris*	USA	ns	4	20	4	38	4	35	4	40	4	50
1487	PI431923	*L.c. culinaris*	USA	ns	4	27	4	47	4	40	4	45	4	45
1488	PI431988	*L.c. culinaris*	USA	ns	4	15	4	53	4	40	4	35	4	45
1489	PI431987	*L.c. culinaris*	USA	ns	4	50	4	45	4	37	4	40	4	45

1490	PI432000	*L.c. culinaris*	USA	ns	4	45	4	40	4	37	4	40	4	43
1491	PI432001	*L.c. culinaris*	USA	ns	4	38	4	47	4	45	4	35	4	35
1492	PI432002	*L.c. culinaris*	USA	ns	4	33	4	38	4	43	4	50	4	35
1493	PI432005	*L.c. culinaris*	USA	ns	4	40	4	57	4	55	4	50	4	50
1494	PI432028	*L.c. culinaris*	USA	ns	4	32	4	45	4	37	4	45	4	38
1495	PI432033	*L.c. culinaris*	USA	ns	4	25	4	50	4	38	4	50	4	48
1497	PI432085	*L.c. culinaris*	USA	ns	4	18	4	48	4	43	4	40	4	43
1498	PI432087	*L.c. culinaris*	USA	ns	4	20	4	40	4	38	4	40	4	30
1499	PI432124	*L.c. culinaris*	USA	ns	4	28	4	38	4	33	4	40	4	13
1500	PI432145	*L.c. culinaris*	USA	ns	4	30	4	43	4	42	4	40	4	35
1501	PI432147	*L.c. culinaris*	USA	ns	4	10	4	53	4	45	4	50	4	50
1502	PI432184	*L.c. culinaris*	USA	ns	ns	ns	4	48	4	42	4	70	4	38
1503	PI432185	*L.c. culinaris*	USA	ns	4	20	4	47	4	43	4	80	4	30
1508	PI432236	*L.c. culinaris*	USA	ns	4	30	4	28	4	35	4	35	4	33
1509	PI432237	*L.c. culinaris*	USA	ns	ns	ns	4	28	4	22	4	40	4	23
1510	PI432245	*L.c. culinaris*	USA	ns	4	50	4	40	4	30	4	60	4	40
1511	PI432259	*L.c. culinaris*	USA	ns	4	10	4	50	4	57	4	60	4	38
1512	PI432271	*L.c. culinaris*	USA	ns	4	35	4	42	4	33	4	25	4	35
1513	PI432286	*L.c. culinaris*	USA	ns	4	35	4	50	4	33	4	50	4	30
1514	PI435954	*L.c. culinaris*	USA	- ns	4	18	4	63	4	47	4	50	4	40
1515	PI451763	*L.c. culinaris*	USA	ns	ns	ns	2	38	2	28	3	45	2	10
1516	PI451766	*L.c. culinaris*	USA	ns	4	14	4	47	4	43	4	45	4	43
1517	PI458503	*L.c. culinaris*	USA	ns	4	4	4	58	4	45	4	50	4	35
1518	PI468899	*L.c. culinaris*	USA	ns	4	1	4	48	4	48	4	60	4	35
1519	PI468900	*L.c. culinaris*	USA	ns	4	4	4	50	4	43	4	50	4	45
1520	PI468902	*L.c. culinaris*	USA	ns	4	20	4	38	4	38	4	50	4	48
1522	PI472122	*L.c. culinaris*	USA	ns	4	30	4	43	4	35	4	40	4	35
1523	PI472136	*L.c. culinaris*	USA	ns	4	30	4	45	4	35	3	30	4	28
1524	PI472137	*L.c. culinaris*	USA	ns	;	0	4	42	4	23	3	15	4	48
1525	PI472143	*L.c. culinaris*	USA	ns	4	15	4	38	4	40	4	50	4	38
1526	PI472161	*L.c. culinaris*	USA	ns	4	30	4	38	4	37	4	60	4	45
1527	PI472175	*L.c. culinaris*	USA	ns	4	5	4	30	4	26	ns	ns	4	28
1528	PI472194	*L.c. culinaris*	USA	ns	4	15	4	35	4	23	4	70	4	35
1529	PI472200	*L.c. culinaris*	USA	ns	4	33	4	47	4	35	4	30	4	30
1530	PI472205	*L.c. culinaris*	USA	ns	4	30	4	53	4	42	4	40	4	40
1531	PI472213	*L.c. culinaris*	USA	ns	4	40	4	40	4	35	4	50	4	40
1532	PI472224	*L.c. culinaris*	USA	ns	4	7	4	38	4	25	4	60	4	25
1533	PI472274	*L.c. culinaris*	USA	ns	4	10	4	54	4	25	4	40	4	33
1534	PI472327	*L.c. culinaris*	USA	ns	4	21	4	35	4	30	4	40	4	33

1535	PI472372	*L.c. culinaris*	USA	ns	4	8	4	28	4	28	4	40	4	30
1536	PI472380	*L.c. culinaris*	USA	ns	4	4	4	47	4	27	4	25	4	30
1537	PI472383	*L.c. culinaris*	USA	ns	4	45	4	38	4	35	4	60	4	33
1538	PI472416	*L.c. culinaris*	USA	ns	4	1	4	43	4	10	4	50	4	35
1539	PI472484	*L.c. culinaris*	USA	ns	4	10	4	40	4	30	4	40	4	43
1540	PI472488	*L.c. culinaris*	USA	ns	4	2	4	28	4	48	4	3	4	50
1541	PI472549	*L.c. culinaris*	USA	ns	4	15	4	28	4	25	4	30	4	25
1542	PI472559	*L.c. culinaris*	USA	ns	4	1	4	37	4	28	4	50	4	40
1543	PI472561	*L.c. culinaris*	USA	ns	4	6	4	48	4	35	4	30	4	35
1544	PI472562	*L.c. culinaris*	USA	ns	4	7	4	25	4	28	4	15	4	28
1545	PI472564	*L.c. culinaris*	USA	ns	4	30	4	45	4	38	4	50	4	40
1546	PI472569	*L.c. culinaris*	USA	ns	4	6	4	43	4	28	4	60	4	48
1547	PI472588	*L.c. culinaris*	USA	ns	4	38	4	33	4	38	4	50	4	45
1548	PI472590	*L.c. culinaris*	USA	ns	4	33	4	37	4	47	4	40	4	40
1549	PI472615	*L.c. culinaris*	USA	ns	4	30	4	45	4	58	4	50	4	45
1550	PI472629	*L.c. culinaris*	USA	ns	4	45	4	50	4	55	4	40	4	20
1552	PI477921	*L.c. culinaris*	USA	ns	ns	ns	3	37	4	15	4	20	;	0
1553	PI486127	*L.c. culinaris*	USA	ns	4	2	3	40	4	35	4	50	2	10
1554	PI486128	*L.c. culinaris*	USA	ns	ns	ns	4	47	4	27	4	40	4	35
1555	PI490288	*L.c. culinaris*	USA	ns	4	8	4	45	4	43	4	70	4	30
1556	PI490289	*L.c. culinaris*	USA	ns	ns	ns	4	47	4	43	4	70	4	35
1557	PI508090	*L.c. culinaris*	USA	ns	ns	ns	4	55	4	47	4	50	4	45
1558	PI518731	*L.c. culinaris*	USA	ns	4	37	4	38	4	38	4	45	4	40
1559	PI518732	*L.c. culinaris*	USA	ns	2	2	1	8	2	5	4	5	;	0
1560	PI518733	*L.c. culinaris*	USA	ns	4-;	6	4	45	4	60	3	35	4	60
1561	PI518734	*L.c. culinaris*	USA	ns	ns	1	;	15	1	7	;	0	;	0
1563	PI533690	*L.c. culinaris*	USA	ns	4	18	4	48	4	48	4	30	4	30
1564	PI533691	*L.c. culinaris*	USA	ns	4	12	4	42	4	47	4	50	4	40
1565	PI533693	*L.c. culinaris*	USA	ns	4	8	4	57	4	50	4	70	4	45
1307	ILWL39	*L.c. odemensis*	Syria	12	4	8	4	30	4	18	4	15	4	1
1314	ILWL 237	*L.c. odemensis*	Syria	30	4	9	4	40	4	8	4	6	4	1
1317	ILWL 261	*L.c. odemensis*	Syria	28	4	1	4	23	4	9	4	21	3	1
1638	PI615677	*L.c. odemensis*	Turkey	ns	ns	ns	4	30	4	20	ns	ns	3	15
1640	PI572361	*L.c. odemensis*	Israel	ns	ns	ns	4	35	4	33	4	40	4	28
1641	PI572362	*L.c. odemensis*		ns	4	5	4	33	4	20	4	30	ns	ns
1608	PI572367	*L.c. orientalis*	Turkey	ns	ns	ns	4	45	ns	ns	ns	ns	ns	ns
1609	PI572369	*L.c. orientalis*	Czech Republic	ns	4	30	4	40	4	30	ns	ns	4	35
1610	PI572370	*L.c. orientalis*	Uzbekistan	ns	ns	ns	4	43	4	25	4	30	4	40

1613	PI572374	*L.c. orientalis*	Iran	ns	4	4	4	45	4	33	4	40	4	50
1614	PI572375	*L.c. orientalis*	Israel	ns	4	2	4	28	4	25	ns	ns	ns	ns
1615	PI572376	*L.c. orientalis*	Turkey	ns	ns	ns	4	30	4	20	ns	ns	ns	ns
1616	PI572379	*L.c. orientalis*	Turkey	ns	ns	ns	4	20	4	35	4	50	4	30
1617	PI572380	*L.c. orientalis*	Iran	ns	4	10	4	25	4	23	4	35	4	10
1618	PI572384	*L.c. orientalis*	Turkey	ns	4	1	4	40	4	43	4	25	4	10
1619	PI572385	*L.c. orientalis*	Turkey	ns	4	1	4	35	4	38	4	15	4	35
1620	PI572386	*L.c. orientalis*	Turkey	ns	4	10	4	38	4	20	4	35	4	23
1621	PI572389	*L.c. orientalis*	Turkey	ns	ns	ns	4	50	4	33	ns	ns	4	23
1622	PI572390	*L.c. orientalis*	Turkey	ns	4	30	4	38	4	38	4	60	4	28
1623	PI572391	*L.c. orientalis*	Cyprus	ns	ns	ns	4	60	4	15	ns	ns	4	38
1624	PI572392	*L.c. orientalis*	Turkey	ns	4	40	4	33	4	37	4	25	4	40
1625	PI572393	*L.c. orientalis*	Turkey	ns	4	40	4	20	4	28	ns	ns	4	33
1626	PI572396	*L.c. orientalis*	Turkey	ns	ns	ns	ns	ns	;	0	ns	ns	ns	ns
1627	PI572397	*L.c. orientalis*	Turkey	ns	4	15	ns	ns	4	15	ns	ns	ns	ns
1628	PI572398	*L.c. orientalis*	Turkey	ns	ns	ns	4	28	4	43	4	40	4	15
1631	PI572406	*L.c. orientalis*	Turkey	ns	ns	0	4	28	4	15	4	50	4	20
1632	PI612249	*L.c. orientalis*	Turkey	ns	4	5	2+	10	4	17	2	5	4	10
1633	PI615668	*L.c. orientalis*	Turkey	ns	4	15	4	47	4	42	4	40	4	40
1636	PI615671	*L.c. orientalis*	Tajikistan	ns	4	10	4	35	ns	ns	ns	ns	4	15
1637	PI615672	*L.c. orientalis*	Tajikistan	ns	4	15	4	30	4	20	ns	ns	4	3
1645	BCU001416	*L.c. orientalis*	Israel	ns	4	30	4	23	4	23	4	70	4	40
1646	BCU001417	*L.c. orientalis*	Israel	ns	4	18	4	25	4	45	4	60	3	10
1647	BCU001418	*L.c. orientalis*	Israel	ns	ns	ns	4	38	4	30	4	50	4	27
1648	BCU001419	*L.c. orientalis*	Israel	ns	4	10	4	35	4	30	4	50	4	35
1649	BCU001420	*L.c. orientalis*	Russia	ns	4	1	4	60	4	25	4	30	ns	ns
1650	BCU001421	*L.c. orientalis*	Cyprus	ns	ns	ns	4	38	4	30	4	40	4	40
1651	BCU001422	*L.c. orientalis*	Israel	ns	4	20	4	40	4	23	4	60	4	50
1652	BCU001423	*L.c. orientalis*	Turkey	ns	4	40	4	30	4	30	4	50	4	25
1308	ILWL40	*L. ervoides*	Syria	0	;	1	1+	6	1	3	2	3	;	0
1312	ILWL 153	*L. ervoides*		10	ns	ns	4	30	4	10	4	30	4	8
1315	ILWL 251	*L. ervoides*	Syria	2	4	2	4	33	4	10	4	30	4	1
1318	ILWL 271	*L. ervoides*	Turkey	10	4	1	ns	ns	4	10	4	47	;	0
1319	ILWL 299	*L. ervoides*	Syria	20	ns	0	4	33	4	1	4	10	4	1
1571	PI572316	*L. ervoides*	Russia	ns	ns	ns	;	0	1	5	ns	ns	ns	ns
1572	PI572317	*L. ervoides*	Italy	ns	4	2	ns	ns	4	15	4	40	ns	ns
1574	PI572319	*L. ervoides*	Serbia	ns	4	1	4	20	3+	20	ns	ns	4	20
1575	PI572320	*L. ervoides*	Serbia	ns	ns	ns	4	30	4	50	ns	ns	ns	ns
1580	PI572325	*L. ervoides*	Croatia	ns	ns	ns	ns	ns	4	30	3	10	ns	ns

1581	PI572326	*L. ervoides*	Serbia	ns	ns	ns	3	15	4	40	ns	ns	4	30
1582	PI572327	*L. ervoides*	Yugoslavia	ns	ns	ns	4	40	4	28	4	10	4	50
1584	PI572329	*L. ervoides*	Israel	ns	4	40	4	30	4	10	4	25	ns	ns
1586	PI572331	*L. ervoides*	Israel	ns	4	20	4	20	3-	25	ns	ns	;	0
1588	PI572333	*L. ervoides*	Turkey	ns	ns	ns	4	15	4	12	ns	ns	4	20
1589	PI572334	*L. ervoides*	Turkey	ns	4	20	ns	ns	4	33	ns	ns	ns	ns
1590	PI572335	*L. ervoides*	Turkey	ns	ns	ns	4	30	4	10	ns	ns	ns	ns
1591	PI572336	*L. ervoides*	Turkey	ns	ns	ns	4	32	4	22	4	50	4	ns
1592	PI572337	*L. ervoides*	Turkey	ns	ns	ns	ns	ns	4	28	ns	ns	4	40
1593	PI572338	*L. ervoides*	Turkey	ns	ns	ns	4	1	ns	ns	4	5	4	10
1594	PI572339	*L. ervoides*	Turkey	ns	4	5	4	40	ns	ns	ns	ns	4	30
1661	BCU001511	*L. lamottei*	Spain	ns	4	1	4	10	4	15	4	5	ns	ns
1665	BCU001527	*L. lamottei*	Spain	ns	4	15	4	25	4	17	4	10	4	25
1302	ILWL26	*L. nigricans*	Syria	10	4	23	4	43	4	30	4	20	4	8
1303	ILWL31	*L. nigricans*	Syria	11	ns	ns	4	19	4	19	4	15	4	20
1311	ILWL38	*L. nigricans*	Syria	1	ns	1	4	13	4	6	4	19	3	2
1595	PI572342	*L. nigricans*	France	ns	ns	0	4	10	4	35	ns	ns	ns	ns
1596	PI572344	*L. nigricans*	Spain	ns	ns	ns	4	20	4	20	ns	ns	ns	ns
1597	PI572346	*L. nigricans*	Italy	ns	ns	ns	4	5	ns	ns	4	30	4	60
1598	PI572348	*L. nigricans*	Yugoslavia	ns	ns	ns	4	50	4	20	ns	ns	ns	ns
1599	PI572349	*L. nigricans*	Serbia	ns	ns	0	1	5	;	0	ns	ns	;	0
1602	PI572352	*L. nigricans*	France	ns	4	15	4	23	4	40	4	30	ns	ns
1603	PI572355	*L. nigricans*	Spain	ns	ns	ns	4	30	ns	ns	4	30	ns	ns
1604	PI572356	*L. nigricans*	Spain	ns	ns	ns	4	18	4	20	4	10	4	8
1607	PI572366	*L. nigricans*	Turkey	ns	ns	ns	4	5	ns	ns	4	20	4	25
1643	BCU001413	*L. nigricans*	Spain	ns	4	30	4	23	4	18	4	15	4	50
1644	BCU001415	*L. nigricans*	Yugoslavia	ns	ns	ns	4	38	4	33	ns	ns	4	50
1653	BCU001424	L. nigricans	Spain	ns	4	33	4	45	4	30	4	40	4	20
1654	BCU001425	*L. nigricans*	Spain	ns	ns	2	4	15	4	33	4	50	4	23
1655	BCU001426	*L. nigricans*	Spain	ns	4	15	4	35	4	25	4	20	ns	ns
1656	BCU001428	*L. nigricans*	Spain	ns	4	23	4	28	1+	15	4	5	2	5
1657	BCU001429	*L. nigricans*	Spain	ns	4	1	4	27	4	20	4	60	4	33
1658	BCU001430	*L. nigricans*	Spain	ns	ns	1	4	5	4	5	ns	ns	4	15
1659	BCU001431	*L. nigricans*	Spain	ns	4	1	4	35	4	35	ns	ns	4	15
1660	BCU001510	*L. nigricans*	Spain	ns	4	5	4	38	4	35	ns	ns	4	33
1662	BCU001524	*L. nigricans*	Spain	ns	ns	ns	4	35	4	28	4	40	4	5
1663	BCU001525	*L. nigricans*	Spain	ns	4	10	4	25	4	20	ns	ns	4	35
1664	BCU001526	*L. nigricans*	Spain	ns	4	14	4	35	4	20	ns	ns	ns	ns
1666	BCU001551	*L. nigricans*	Spain	ns	4	5	4	27	4	12	4	35	4	5

1667	BCU001577	*L. nigricans*	Spain	ns	4	40	ns	ns	4	40	ns	ns	4	28
1668	BCU001887	*L. nigricans*	Spain	ns	4	35	4	43	4	25	4	30	ns	ns
1669	BCU001890	*L. nigricans*	Spain	ns	4	20	4	18	4	23	ns	ns	4	15
1670	BCU001894	*L. nigricans*	Spain	ns	4	23	ns	ns	4	25	ns	ns	4	5
1671	BCU001896	*L. nigricans*	Spain	ns	4	20	4	15	4	13	ns	ns	ns	ns
1672	BCU001900	*L. nigricans*	Spain	ns	4	33	4	35	4	20	4	30	4	18
1672	BCU001900	*L. nigricans*	Spain	ns	4	33	4	35	4	20	4	30	4	18
1673	BCU001901	*L. nigricans*	Spain	ns	4	15	4	15	4	7	ns	ns	ns	ns
